# Correction: Well protected SmCo nanoclusters: fabrication and transformation to single crystals

**DOI:** 10.1039/c7ra90117h

**Published:** 2018-01-16

**Authors:** Nadeem Abbas, Jian-zhong Ding, J. Ping Liu, Juan Du, Wei-xing Xia, A-ru Yan, Fang Wang, Jian Zhang

**Affiliations:** CAS Key Laboratory of Magnetic Materials and Devices, Ningbo Institute of Materials Technology and Engineering, Chinese Academy of Sciences Ningbo 315201 China zhangj@nimte.ac.cn; Zhejiang Province Key Laboratory of Magnetic Materials and Application Technology, Ningbo Institute of Materials Technology and Engineering, Chinese Academy of Sciences Ningbo 315201 China; University of Chinese Academy of Sciences 19 A Yuquan Rd., Shijingshan District Beijing 100049 China; Department of Physics, University of Texas at Arlington Arlington Texas 76019 USA; Ningbo University of Technology Ningbo Zhejiang 315211 China

Correction for ‘Well protected SmCo nanoclusters: fabrication and transformation to single crystals’ by Nadeem Abbas *et al., RSC Adv.*, 2017, **7**, 51695–51701.

The authors regret that an institution was missing in the original article. The correct author affiliation list in which author affiliation *c* has been added for Nadeem Abbas is presented herein.

The Royal Society of Chemistry apologises for these errors and any consequent inconvenience to authors and readers.

## Supplementary Material

